# Supplementary material for: Utility of Kolmogorov complexity measures: Analysis of L2 groups and L1 backgrounds
Source: PLoS One. 2024 Apr 18;19(4):e0301806. doi: 10.1371/journal.pone.0301806 (PMC11026146; doi:10.1371/journal.pone.0301806)
Supplement: Attachment — Submitted filename: comments.pdf [file pone.0301806.s001.pdf]

This study examined the differences in Kolmogorov complexity between L2 proficiency levels across distinct languages and across different L1s. I think it is interesting and significant to consider the potential influence of L1 background on L2 writing complexity. However, I have some main concerns at both of the conceptual and methodological levels.

Regarding RQ1, which aims to compare the differences in Kolmogorov complexity across different L2 proficiency levels (A1 and B1) in four languages (German, Italian, Czech, and English), I find the rationale for this comparison unclear. It is evident that there would be cross-linguistic differences in Kolmogorov complexity due to variations in language structures and features among the four languages. However, the significance of this comparison in the context of L2 proficiency development remains ambiguous.

Additionally, I don't see the rationale for discussing the cross-linguistics differences with the topics, settings, and writing genres uncontrolled. I suggest a clearer explanation of the rationale behind RQ1 and a more explicit description of the methodology to highlight the study's significance.

Moving on to RQ2, which explores the impact of L1 background on L2 English writing Kolmogorov complexity, I question the choice of using Kolmogorov complexity metrics instead of traditional syntactic complexity metrics. Given that English is the target language for RQ2, it seems more appropriate to utilize the traditional commonly used syntactic complexity metrics for this analysis. Furthermore, the discussion of L1 effects on L2 writing complexity lacks depth and fails to fully explore the potential implications of the findings based on the diverse L1 backgrounds examined. The methodology lacks details regarding the selection criteria for written essays, including topics, settings, and writing genres, which are crucial factors that could influence the observed differences in Kolmogorov complexity. I recommend a thorough discussion of the potential L1 effects based on the diverse L1 backgrounds and a clearer description of the essays selection process from the corpus.

Additionally, I suggest updating the literature review to include recent studies on syntactic complexity validation that are relevant to the study's objectives. For example, Xu, T. S., Zhang, L. J., & Gaffney, J. S. (2023). A multidimensional approach to assessing the effects of task complexity on L2 students' argumentative writing. *Assessing Writing*, 55, 100690, 1-16. https://doi.org/10.1016/j.asw.2022.100690 By incorporating these recent findings, the study can provide a more comprehensive review of the existing literature and strengthen its theoretical framework.

Finally, in the conclusion, there is a need for expansion, as there appears to be a lack of discussion regarding the significance and implications of the study's findings.
