# Supplementary material for: Utility of Kolmogorov complexity measures: Analysis of L2 groups and L1 backgrounds
Source: PLoS One. 2024 Apr 18;19(4):e0301806. doi: 10.1371/journal.pone.0301806 (PMC11026146; doi:10.1371/journal.pone.0301806)
Supplement: Attachment — Submitted filename: Response to Reviewers.docx [file pone.0301806.s003.docx]

I thank the reviewers for their very helpful comments. In the following, I included each comment followed by my reply.

1. Regarding the Method-corpus section, the author introduced the corpus as "...,whereas EFCAMDAT also involved persuasive texts (e.g., selling items in an online auction)." The statement is unclarified. I suggest further explain whether EFCAMDA exclusively comprised persuasive genre texts or if the author specifically selected persuasive texts from a diverse range of genres.

Thank you for pointing out this point. I clarified this part as follows:

“…, whereas EFCAMDAT largely comprised persuasive texts (e.g., selling items in an online auction). These were the main genres in each corpus.”

2. In the third paragraph of the Method-corpus section, the phrase "For MERLIN, only texts rated A1 and B1 were selected as the other CEFR levels were not equally represented across L2" should be revised for better understanding.

I modified the sentence as follows:

“For MERLIN, this study selected texts with A1 and B1 ratings because texts with higher or lower CEFR ratings were not equally represented across L2 in this corpus.”

I hope it’s easier to read and understand now.

3. Also in the third paragraph of the Method-corpus part, the author mentioned “Since EFCAMDAT is semi-longitudinal (Shatz, 2020), texts written by the same L2 learner were removed to reduce idiosyncratic complexity features.” Could you further explain the removal of texts written by the same L2 learner? How to remove? To remove identical essays by individual writers or all essays produced by a single writer?

Thank you for sharing this suggestion. I revised this part as follows:

“EFCAMDAT is semi-longitudinal and contains several texts written by the same L2 learner (Shatz, 2020). This study selected only one text per L2 learner to reduce idiosyncratic complexity features.”

4. I still think it is necessary to include the traditional measures to account for L1 based differences (RQ2). However, I did not locate this point in the limitation section, which I think should be explicitly addressed in the manuscript.

I agree with the reviewer. This point is now mentioned in the limitation section as follows:

“Finally, the inclusion of traditional measures along with Kolmogorov complexity (e.g., Wang et al., 2022) could foster a better understanding of L1 based differences.”

5. Please carefully proofread the manuscript.

Thank you for your suggestions. The manuscript has been proofread for grammar and word choice. I hope it has much improved now.
